# Are there socioeconomic disparities in geographic accessibility to community first responders to out-of-hospital cardiac arrest in Ireland?

**DOI:** 10.1016/j.ssmph.2022.101151

**Published:** 2022-06-22

**Authors:** Siobhán Masterson, Conor Teljeur, John Cullinan

**Affiliations:** a Discipline of General Practice, National University of Ireland Galway, Ireland and Clinical Directorate, National Ambulance Service, Limerick, Ireland; b Department of Public Health & Primary Care, Trinity College Dublin, Ireland; c Discipline of Economics, JE Cairnes School of Business and Economics, National University of Ireland Galway, Galway, Ireland

**Keywords:** Out-of-hospital cardiac arrest, Resuscitation, First responders, Volunteerism, Community, Socioeconomic status

## Abstract

Out-of-hospital cardiac arrest (OHCA) is a leading cause of death worldwide. Without appropriate early resuscitation interventions, the prospect of survival is limited. This means that an effective community response is a critical enabler of increasing the number of people who survive. However, while OHCA incidence is higher in more deprived areas, propensity to volunteer is, in general, associated with higher socioeconomic status. In this context, we consider whether there are socioeconomic disparities in geographic accessibility to volunteer community first responders (CFRs) in Ireland, where CFR groups have developed organically and communities self-select to participate. We use geographic information systems and propensity score matching to generate a set of control areas with which to compare established CFR catchment areas. Differences between CFRs and controls in terms of the distribution of catchment deprivation and social fragmentation scores are assessed using two-sided Kolmogorov-Smirnov tests. Overall we find that while CFR schemes are centred in more deprived and socially fragmented areas, beyond a catchment of 4 min there is no evidence of differences in area-level deprivation or social fragmentation. Our findings show that self-selection as a model of CFR recruitment does not lead to more deprived areas being disadvantaged in terms of access to CFR schemes. This means that community-led health interventions can develop to the benefit of community members across the socioeconomic spectrum and may be relevant for other countries and jurisdictions looking to support similar models within communities.

## Highlights

• Out-of-hospital cardiac arrest (OHCA) is a major cause of unexpected death.
• OHCA is more prevalent in deprived areas and community response is key for survival.
• Irish community first responders (CFRs) self-select to participate in CFR schemes.
• We consider if there are socioeconomic disparities in geographic access to CFRs.
• Self-selection does not result in deprived areas having worse access to CFR schemes.

## 1 Introduction

Cardiac arrest occurs when the heart stops beating or beats in a manner that prevents blood from circulating around the body (Paradis et al., 2007). Out-of-hospital cardiac arrest (OHCA), defined as an unexpected cardiac arrest event that occurs in a location other than an acute hospital, is the third leading cause of death in Europe (Gräsner et al., 2020). While death from OHCA is frequent, it is not inevitable, particularly if treatment is initiated immediately or within minutes of collapse. As a result, the ‘chain of survival’ concept was approved by the American Heart Association (AHA) in 1990 (Cummins et al., 1991) and is used internationally to describe the series of resuscitation interventions required to restore consciousness or other signs of life in an OHCA patient. It includes four key ‘links’, namely (i) early recognition of OHCA and immediate call for help to the Emergency Medical Services (EMS), (ii) immediate, high-quality cardiopulmonary resuscitation (CPR), (iii) defibrillation within minutes of collapse, and (iv) effective advanced EMS and post-resuscitation care.

In a recent iteration, the importance of the first two links in the chain of survival (call to emergency services and immediate, high quality cardiopulmonary resuscitation) were emphasised, given they have the greatest potential to impact on survival (Deakin, 2018). Importantly, in the case of OHCA, their success is invariably dependent on the community response, rather than the EMS. The third link, defibrillation, is also independently associated with survival. It involves stopping ‘fibrillation’ of the heart by administering a controlled electric shock, in order to allow restoration of the normal rhythm. Notably, however, the benefit of defibrillation reduces as the interval from time of emergency call to defibrillation increases (Hansen et al., 2015; Kiyohara et al., 2019). Ireland was to the forefront in making defibrillation available in the prehospital setting in the 1960s, first with the invention of a mobile defibrillator in Belfast (Adgey et al., 1969) and then with the provision of prehospital defibrillation by paramedics in Dublin (Gearty et al., 1971). Automated external defibrillators (AEDs) have become increasingly available in the community and defibrillation before EMS arrival is consistently associated with improved OHCA survival (Blom et al., 2014; Hallstrom et al., 2004; Nielsen et al., 2013; Wissenberg et al., 2013). This implies that a critical advantage of so-called ‘first responders’ lies in their potential to shorten the interval from collapse to first defibrillation.

Various first responder models involving citizens, firefighters, police, and off-duty EMS personnel have been developed across many countries to support and improve the community-based first response to OHCA (Oving et al., 2019). Citizen responders are common across European countries, but the model of a citizen-based ‘community first responder’ (CFR) scheme is most closely linked to Ireland and the United Kingdom (UK). In Ireland, the setting for this study, the provision of emergency first response by volunteer CFR schemes was established over 25 years ago and the role and value of CFRs was formally recognised in the Report of the Task Force on Sudden Cardiac Death (Department of Health and Children, 2006). In particular, it recommended the enhancement of first responder programmes to reduce response times in the event of cardiac arrest. The potential impact of extending the scope and number of CFR schemes was subsequently assessed in the ‘Lightfoot Report’, which estimated that ‘optimal CFR contribution’ could significantly improve response times to life-threatening emergencies in Ireland (Lightfoot Solutions UK Limited, 2015).

Health service provision is usually planned by health government agencies. Even where local health agencies or communities identify a local need, approval and funding for health service provision is usually controlled at a national level. In contrast, communities involved in the Irish CFR scheme are self-selecting. However, the propensity for community volunteerism has long been understood to be associated with higher personal and area-level socioeconomic status (Bell & Force, 1956; Niebuur et al., 2018; Youssim et al., 2015). For example, in 2016 the European Quality of Life Survey found that volunteering rates were greater when people were employed, had high educational attainment, and high income (Eurofound, 2017). More recently, in 2019, the UK National Council for Voluntary Organisations found that formal volunteering was more common among people who lived in the least deprived areas when compared to those who lived in the most deprived areas (29% vs. 14%) (The National Council for Voluntary Organisations, 2020). This is particularly relevant when considering services targeting OHCA, since previous research has found that lower socioeconomic status is associated with both a greater incidence of OHCA and poorer survival (Hallstrom et al., 1993; Sasson et al., 2012). Indeed, recent evidence from Ireland found a statistical association between greater area-level deprivation and increased OHCA incidence (Masterson et al., 2018).

Given all this, it is important to highlight that CFR schemes in Ireland rely on community self-selection and volunteerism. Thus, since OHCA incidence and outcomes are associated with lower socioeconomic status, while volunteerism is generally associated with higher socioeconomic status, it is important to consider if the specific nature and features of the Irish scheme have led to socioeconomic disparities in geographic accessibility to CFRs. This would be particularly problematic if CFR schemes were less likely to be located in more deprived areas, where the risk and incidence of OHCA tends to be greater, since it would imply that the ‘organic’ nature and development of CFR schemes is resulting in a sub-optimal geographic distribution of healthcare services. In this context, this paper considers the socioeconomic profile of areas with geographic accessibility to CFRs in Ireland and compares this to similar ‘control’ areas that have no accessibility. In particular, it investigates the presence or not of differences in both area-level deprivation and social fragmentation between CFR and control catchments in order to investigate if there are socioeconomic disparities in geographic accessibility to CFRs.

The paper is structured as follows: Section 2 describes the setting for our analysis, Section 3 our materials and methods, while Section 4 presents our key results and findings. Finally, Section 5 discusses the implications of our research and concludes.

## 2 Setting

There are in the region of 5,000 OHCAs in Ireland each year, of which approximately 2,200 with resuscitation attempts are recorded in the OHCA Registry (Irish National Out-of-Hospital Cardiac Arrest Register, 2019). In 2019, 67% of OHCA patients were male and the median age of patients was 68 years (interquartile range: 54–79 years). The majority of incidents occurred in an urban area, though the incidence was similar in both urban and rural areas (53 vs. 50 OHCA patients per 100,000 population respectively). Most events occurred at home (68%) and the vast majority of patients had bystander CPR attempted (84%). Only 7% of patients had defibrillation attempted before the arrival of the EMS and in 2019 a total of 190 people survived an OHCA event. From the perspective of the impact of community intervention, 49% of survivors had defibrillation attempted before EMS arrival.

As part of the health system response in Ireland, CFRs have been a feature of the health service for over 25 years. In 2004, the sudden death of a young high profile athlete raised the issue of community first response in the media and contributed to renewed interest in the establishment of CFR schemes (Irish Independent, 2016). In 2008, the Prehospital Emergency Care Council (PHECC) has established PHECC CFR education and training standards and the completion of a course which meets these standards is mandatory prior to becoming a CFR (Prehospital Emergency Care Council (PHECC) (2020). Schemes have been established with the assistance of ambulance personnel, community leaders, and organisations such as CFR Ireland and the Irish Heart Foundation. At the time of this study, there were 222 active CFR groups in Ireland that were linked to the National Ambulance Service (NAS) and these are included in our analysis. Previous research has estimated that Irish CFRs have the potential to reach approximately one million additional citizens before the ambulance service and within a timeframe where defibrillation is likely to be effective (Barry et al., 2018).

In terms of the process involved in establishing a CFR scheme, communities self-select into the scheme and must first express an interest before Community Engagement Officers from the NAS offer assistance. Community Engagement Officers provide support during the CFR scheme set up and certify that necessary training and health and safety requirements are met before the CFR scheme can be alerted to an emergency call. They also provide ongoing support to existing groups in the form of training events, regular visits, and contacts. However, while Community Engagement Officers must certify that a CFR scheme is fully prepared to respond to emergency calls, individual CFR schemes remain responsible for: recruiting members; financing and purchasing equipment including defibrillators; and, ensuring that the clinical training, health and safety requirements, and vehicle insurance arrangements of CFR scheme members are met at all times.

CFR schemes are linked with the NAS Emergency Operations Centre (NEOC) and are alerted in the event of a suspected OHCA within a designated area. The radius of the area covered may vary, depending on population density and geography, but is usually 5–10 kms. In the event that a cardiac arrest is suspected, and if the location is within the radius of the CFR scheme coverage, an automated text alert is sent from NEOC to the CFR scheme. If the CFR scheme members accept the call and travel to the scene, they are required to perform CPR and attempt defibrillation until the patient recovers or until the arrival of the statutory ambulance services. All CFR schemes are equipped with defibrillators, which they bring directly to the event location. It is of note that this model of direct dispatch has been shown to decrease time-to-defibrillation when compared to a model where the first responder must first access the closest public access AED (Auricchio et al., 2019).

In terms of the population and geographic context for this study, Ireland has a population of over 5 million people, with approximately 37% of people living in rural areas (Central Statistics Office, 2019). The smallest legally defined area in Ireland is the electoral division (ED), of which there are 3,409. The population and geographic coverage of EDs varies considerably with a mean population of 1,397 (range: 66 to 38,894). Since 2011, census outputs are also reported for 18,641 subdivisions of EDs, called small areas, which have a mean population of 255 (range: 50 to 1,629).

## 3 Materials and methods

### 3.1 Data

Our analysis uses data from a range of sources. First, when a CFR scheme is established, the CFR scheme coordinator advises the NAS of the address that represents the most central location in their catchment area. This address is used as a centroid to generate a radius within which the CFR scheme will be alerted to cardiac arrest calls by NEOC. The centroid for each CFR group was supplied by the NAS, which allowed us to map the catchment areas of all 222 active CFR groups in Ireland.

Second, in order to consider the relative socioeconomic profile of these CFR catchment areas, it was necessary to develop a set of control group catchment areas. To generate potential control group centroids, two house points were randomly selected from each ED to give a total of 6,818 potential locations. House points were used to ensure that centroids were in habitable locations and close to the road network. By sampling from all EDs, there was unbiased coverage of socioeconomic conditions.

Third, to consider the socioeconomic profile of CFR catchment and control areas, measures of area-level deprivation and social fragmentation were used. Deprivation was defined using an index designed for health services research (Teljeur et al., 2019). It is based on four indicators from the 2016 census of population that are combined using principal components analysis (PCA), namely: unemployment; low social class; car ownership; and, local authority housing. The potential influence of social capital was also explored using an index of social fragmentation (Congdon, 2004). The index was also computed by applying PCA, this time to four census variables, namely: unmarried; single person households; rented accommodation; and, moved in last 12 months. This index was intended to capture populations in flux and which are likely to have diminished social capital as a result. Both indices were computed at small area level and expressed as a continuous variable. The Moran's I is 0.569 for deprivation and 0.613 for social fragmentation, indicating the relatively high degree of spatial autocorrelation in these indicators.

Finally, the locations of ambulance stations and hospital emergency departments (ED) with 24/7 cover were geocoded. This allowed us to calculate travel time measures from the centroids of CFR and control catchments to these facilities.

### 3.2 Catchment generation

Catchment areas were defined based on small areas within a specified travel time of CFR and control centroids. Road network data for the whole island of Ireland were accessed from OpenStreetMap. Travel time calculations were undertaken using OSRM, accessed through R (Giraud, 2020; R Development Core Team, 2020). For the main analysis it was assumed that the effective coverage of a CFR group would be up to 8 min from the centroid based on travel by private car. The decision to use an 8-min catchment area was based on the response time key performance indicator for Irish ambulance services which requires that “patients with life-threatening cardiac or respiratory arrest incidents are responded to by a first responder … in 7 min and 59 s or less in 75% of all cases” (Health Information and Quality Authority, 2012). The characteristics of the small areas within a catchment were used to calculate population-weighted catchment-level characteristics (e.g., mean deprivation score).

### 3.3 Propensity score matching

To select matched controls from the full set of 6,818 potential locations described above, propensity score matching was used. Scores were computed on the basis of 9 variables: distance to the nearest ambulance station; distance to the nearest hospital ED; distance to the nearest city; distance to the nearest town; distance to the nearest village (classified into ‘near’ and ‘remote’); area covered by the catchment (km^2^); the population of the catchment; and, the population density of the catchment. Cities, towns, and villages were defined on the basis of an urban-rural index (Teljeur & Kelly, 2008).

Propensity score matching was undertaken in R using the MatchIt package (Stuart et al., 2011). Both nearest neighbour and optimal methods were used. Distance metrics were calculated on the basis of both standard logit and Mahalanobis distance. For the nearest neighbour method, a range of caliper values were tested: 0, 0.1, and, 0.25. In all analyses a one-to-one ratio was used to generate 222 matched controls. As a sensitivity analysis, matching ratios of two, three and four to one were also tested for all eight model specifications. To compare the outputs of the various approaches to propensity score matching, the standardised differences in the matching variables between CFRs and controls were assessed using a Chi-square test (Baser, 2006). A statistically significant result (p ≤ 0.05) would indicate that at least one of the variables included in the model was creating an imbalance between CFRs and controls. The matching models were also compared using five balance checking criteria to investigate evidence of selection bias (Baser, 2006).

### 3.4 Statistical analysis

The difference between CFRs and controls in terms of the distribution of catchment deprivation and social fragmentation scores was assessed using a two-sided Kolmogorov-Smirnov test. The test is non-parametric and can distinguish between distributions that have the same mean but a different variance. Because some CFRs were in close proximity, the catchments in some cases overlapped. The degree of overlap between catchments in terms of shared small areas was calculated to compare CFRs to matched controls. Overlap was expressed as a proportion of small areas in a catchment that are also in another catchments.

With increasing catchment size, the increasing heterogeneity of small areas included in catchments would likely lead to the mean socio-economic conditions converging towards the national mean. To test whether this was an issue, the distribution of catchment deprivation and social fragmentation was also calculated for random selections of control catchments.

As the propensity score matching was based on nine variables, there was a risk of over-matching. To explore this issue and the impact of excluding different variables, a sensitivity analysis using a leave-one-out approach was used.

## 4 Results

Eight different configurations of the propensity score matching algorithm were tested. The outputs of the optimal matching method indicated that there was an imbalance in at least one variable – see Table 1. At a catchment distance of 8 min, the lowest Chi-square value was for nearest neighbour matching with a caliper of 0.1, using either logit or Mahalanobis distance. On inspection of the balancing checking criteria, it was felt that the model using Mahalanobis distance was marginally better and that model configuration is used for subsequent reporting here (see supplementary appendix for details of balance checking criteria).

**Table 1:** Chi-square test results for eight propensity score models tested.

Matching method	Distance measure	Caliper	Chi-square	p value
Nearest neighbour	logit	None	3.40	0.946
Nearest neighbour	logit	0.10	1.85	0.994
Nearest neighbour	logit	0.25	2.96	0.966
Nearest neighbour	Mahalanobis	None	1.93	0.993
Nearest neighbour	Mahalanobis	0.10	1.87	0.993
Nearest neighbour	Mahalanobis	0.25	1.90	0.993
Optimal	logit	None	246.20	0.000
Optimal	Mahalanobis	None	262.99	0.000

Note: All Chi-square tests had nine degrees of freedom.

The matched controls demonstrated a good balance across the matching variables – see Table 2. However, it should be noted that CFR groups were more likely to be centred in a town or village area, and less likely to be centred in a rural area, than the matched controls. The average overlap between CFR catchments was 0.22 (Interquartile range (IQR): 0.00 to 0.38) and the equivalent for matched controls was 0.24 (IQR: 0.00 to 0.44).

**Table 2:** Characteristics of CFR and control catchments.

Characteristic	CFR catchments	Matched control catchments	All control catchments
*Area type, n (%)*			
City	16 (7%)	14 (6%)	954 (14%)
Town	62 (28%)	45 (20%)	586 (9%)
Village	39 (18%)	25 (11%)	532 (8%)
Rural	105 (47%)	138 (63%)	4,746 (70%)
*Variables used for propensity score matching, mean (SD)*			
Area (km^2^)	59.1 (30.5)	57.6 (28.1)	51.0 (27.2)
Population	10,018 (21,412)	7,867 (19,639)	17,609 (40,032)
Population density (persons/km^2^)	240.9 (614.8)	186.9 (558.1)	490.9 (1,196.6)
Travel time to nearest ambulance station (minutes)	16.7 (18.0)	16.7 (17.8)	15.9 (9.6)
Travel time to nearest hospital ED (minutes)	34.6 (26.2)	34.4 (25.8)	32.0 (19.4)
Travel time to nearest city (minutes)	59.1 (57.2)	58.7 (56.7)	56.2 (44.8)
Travel time to nearest town (minutes)	15.9 (22.8)	15.9 (22.3)	15.1 (10.3)
Travel time to nearest village (near) (minutes)	19.5 (23.5)	19.8 (23.0)	18.3 (13.2)
Travel time to nearest village (remote) (minutes)	23.1 (19.3)	23.2 (18.8)	25.9 (58.6)

Abbreviations: SD = standard deviation; ED = emergency department.

**Fig. 1:**
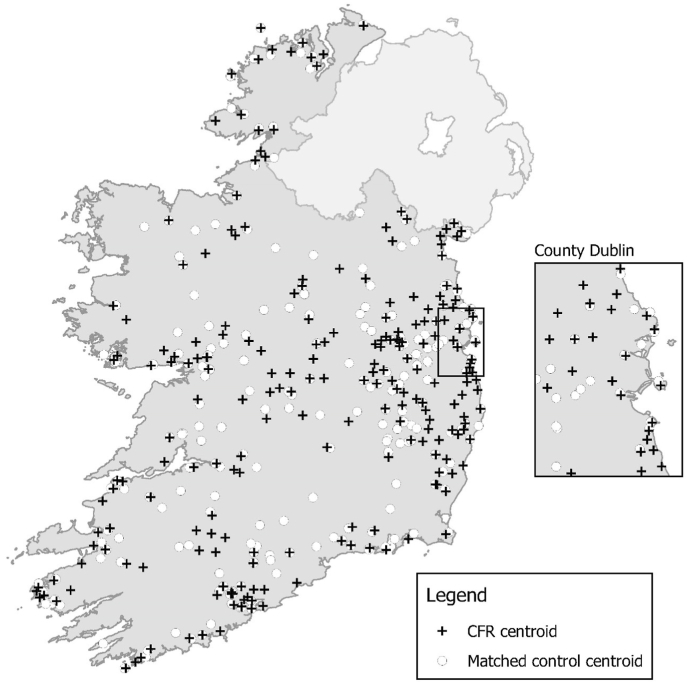
Map of Community First Responder and control locations based on 8 min catchments.

Fig. 1 presents a map of CFR and matched control locations based on 8-min catchments. It clearly shows that the geographic distribution of CFR schemes in Ireland is far from uniform and that there are large areas of the country without a CFR scheme. While nationally the correlation between deprivation and social fragmentation is 0.514, the correlation was 0.670 for CFR groups and 0.542 for matched controls. Overall 21% of CFR centroids (n = 46) were within 8 min travel time of an ambulance station, compared with 20% of matched controls (n = 45) and 23% of all controls. The mean straight line distance from a CFR centroid and its nearest neighbouring CFR centroid was 8.5 km (range: 0.6–32.5 km). The equivalent for the matched controls was 8.8 km (range: 0.3–39.2 km).

**Fig. 2:**
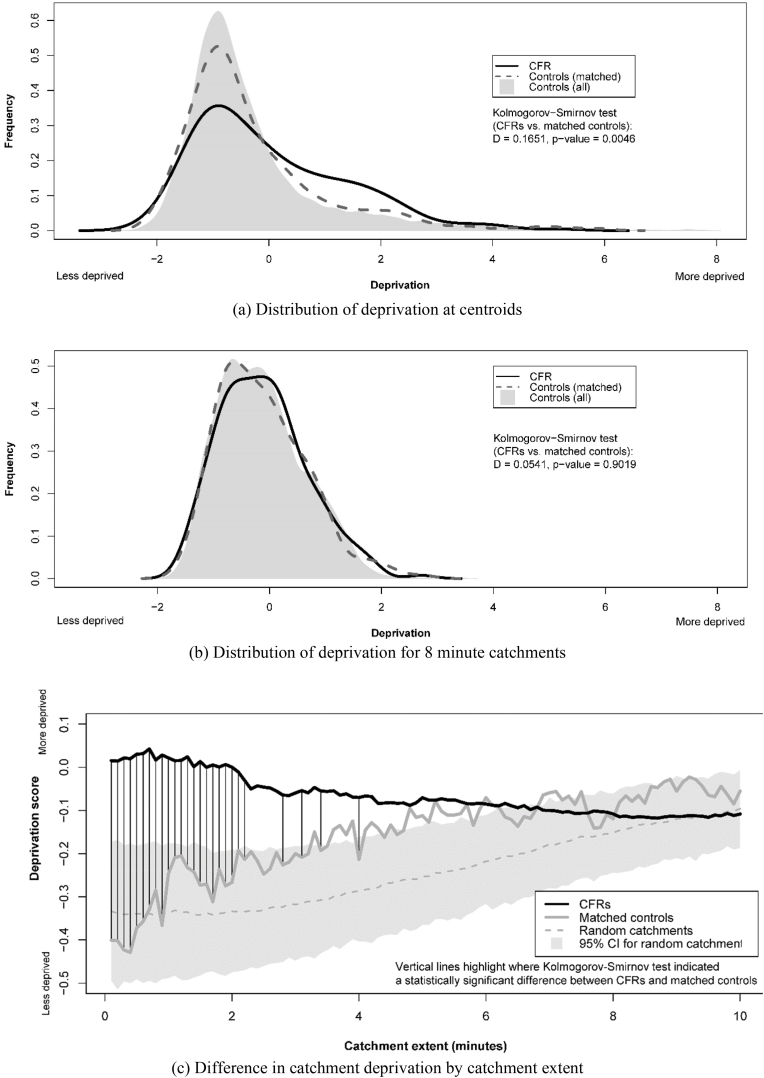
Distribution of catchment deprivation scores.

In terms of deprivation, CFRs were more deprived at their catchment centroids than the matched controls (D = 0.17, p = 0.005) but not for 8 min catchments (D = 0.05, p = 0.902) (Fig. 2(a) and (b)). The difference in catchment deprivation between CFRs and matched controls was significant to a catchment of 2.2 min, then occasionally significant to a catchment of 4.0 min, and not thereafter (Fig. 2(c)).

**Fig. 3:**
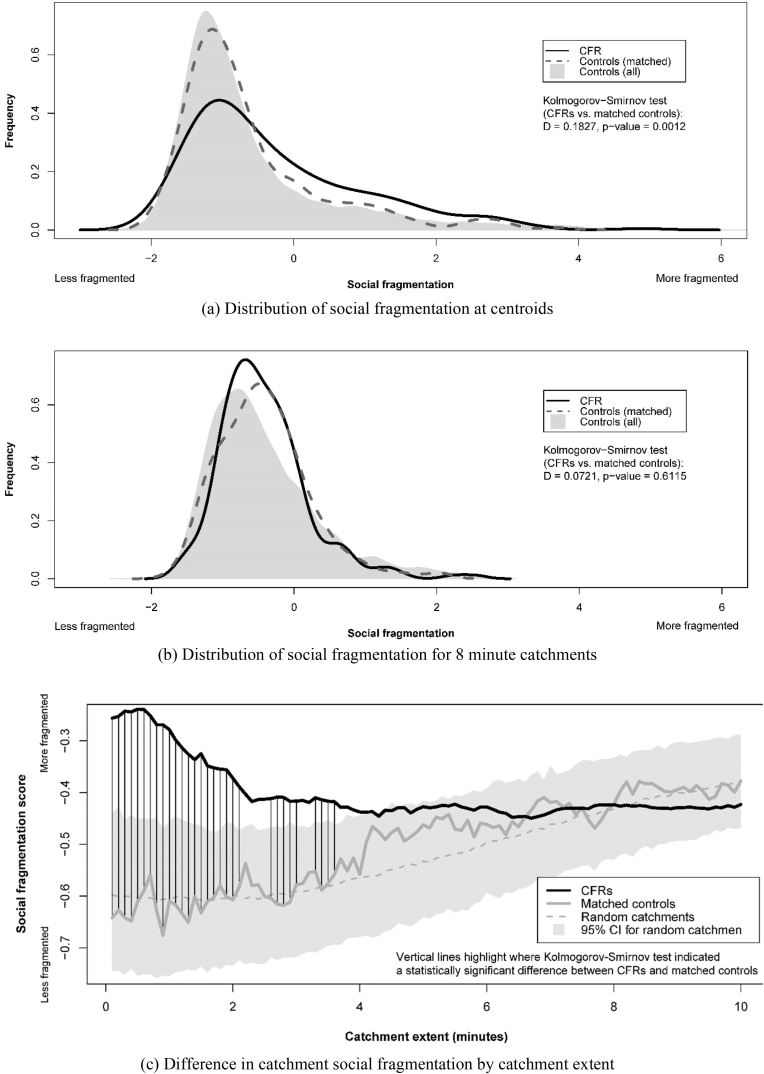
Distribution of catchment social fragmentation scores.

Similarly for social fragmentation, CFRs were more fragmented at their catchment centroids than the matched controls (D = 0.18, p = 0.001) but not for 8 min catchments (D = 0.07, p = 0.612) (Fig. 3(a) and (b)). The difference between CFRs and matched controls in catchment social fragmentation is significant to a catchment of 2.1 min, then sporadically significant to a catchment of 3.6 min, and not thereafter (Fig. 3(c)).

By comparison with control catchments generated by random selection rather than propensity score matching, the difference between the CFR catchments and the random selections decreases with increasing catchment extent. At a catchment extent of 5.8 min, there is a less than 50% chance that the difference in deprivation between the CFR catchments and a random selection will be statistically significant. At an extent of 6.7 min, there is a less than 50% chance that the difference in social fragmentation between the CFR catchments and a random selection will be statistically significant.

A sensitivity analysis was undertaken to test the impact of leaving out each matching variable in turn on the findings. For both deprivation and social fragmentation, omission of either area, population or population density had an impact on the results. With omission of area, there was almost no catchment extents at which CFRs were significantly different to matched controls for deprivation or social-fragmentation. For population and population density, the extent to which significant differences were observed was reduced. However, in all cases the quality of the matching was based on the Chi-square tests. On inspection, the matched controls areas tended to cover a larger geographic area and have a notably lower population density than the CFR catchments. The inclusion of additional controls for each case did not improve the model fit, and generally resulted in increased variance observed in the matching variables.

## 5 Discussion

The aim of this study was to investigate if there are socioeconomic disparities in geographic accessibility to CFR groups in Ireland. We used a propensity score matching approach to select relevant control areas to compare with the established CFR catchments in terms of area-level deprivation or social fragmentation. The analysis provides evidence that while CFRs may be centred in areas that are, on average, more deprived and more socially fragmented, beyond a catchment extent of 4 min this is no longer apparent. Given the heterogeneity in deprivation at larger catchment extents, we would not expect to observe a difference at extents of more than 6 min.

Given that OHCA incidence tends to be associated with lower socioeconomic status, this is a desirable finding. However, these differences in the socioeconomic status of CFR and matched control catchment centroids may be partly explained by the sampling method. The full list of control areas was based on two randomly sampled points for each of the 3,409 EDs in Ireland. Based on the distribution of centroids by area type (Table 2), it can be seen that CFRs and control areas were located in quite different areas. While 14% of control areas were centred in city EDs, only 7% of CFRs are. The distribution of the matched controls areas was closer to that of the CFR centroids, but there was still over-representation of rural EDs. The differences in socioeconomic status at the centroids may be partly explained by the different distribution by area type, although it should be noted that the large set of control points to sample from meant that a matching distribution by area type could have been achieved. The fact that there was a difference by area type between CFRs and matched controls, particularly in terms of the seeming under-representation of rural EDs, may suggest that an economy of scale, or minimum population, is required to generate sufficient volunteers or resources to support the establishment of a CFR.

Overall, the fact that CFR centroids are more deprived and more socially fragmented is not particularly important, as in reality CFR members will respond from their homes. Nonetheless, it is interesting that centroids tend to be more deprived and socially fragmented, as it shows that the establishment of CFRs in Ireland is not centred on more socioeconomically advantaged areas. If anything, it is likely the reverse. So even if there is a propensity for higher socioeconomic status individuals to volunteer, this does not result in less chance of CFR coverage for areas with lower socioeconomic status.

Our findings have important implications for the design and development of CFR schemes, both in Ireland and more generally. As discussed, voluntary first response is different to other health services in terms of its organic, rather than planned, development and, in particular, in relation to the way local communities self-select into the scheme. In addition, our results suggest that socioeconomically disadvantaged areas do not lose out as a result of the volunteering element of the Irish scheme.

Early community intervention is the key to increasing survival after OHCA and CFRs play a critical role in tackling OHCA. For example, clinical trials have shown that first responders can increase rates of CPR and defibrillation before EMS arrival (Barry et al., 2019), while observational studies have suggested an important role for community-based AED use in increasing OHCA survival (Bækgaard et al., 2017). In a retrospective evaluation of OHCA data from North Carolina, Hansen and colleagues showed that first responders were responsible for the majority of instances (51.8%) of ‘early’ defibrillation (i.e. time from emergency call to defibrillation within 5 min) (Hansen et al., 2015). The Copenhagen Oslo STockholm Amsterdam (COSTA) Group reported on the survival status of 22,453 patients and observed that of the 2,957 patients who survived to at least 30 days post-event, 454 (20%) were defibrillated by a first responder AED (Zijlstra et al., 2018). Studies from The Netherlands and Sweden have also demonstrated significant reductions in time-to-defibrillation when dispatched first responders were compared to the EMS (Claesson et al., 2017; Zijlstra et al., 2014). Our results suggest that communities in Ireland are not disadvantaged by socioeconomic status in relation to such benefits.

Nonetheless, despite our findings in relation to the lack of problematic socioeconomic disparities, it is worth noting that our mapping of CFRs in Ireland shows there are large areas of the country currently with no CFR coverage. This suggests that while the organic development of CFR schemes has been successful in avoiding potential socioeconomic disadvantage in coverage, there likely remains a need for increased coverage of CFR schemes in Ireland overall. Previous research in Scotland has suggested that CFR schemes that are supported are a sustainable model type once established (Farmer et al., 2015). Future research could examine the individual and community-level motivations for establishing a scheme and whether clustering is a feature of CFR scheme development.

Finally, it is important to acknowledge some caveats associated with our results and findings. First, a central feature of the analysis was the comparison of the socioeconomic status of CFR and matched control catchments as defined by our choice of catchment extent. The use of an 8-min catchment was a pragmatic choice based on a response time key performance indicator (Health Information and Quality Authority, 2012). The choice of catchment size is clearly important as population heterogeneity increases with distance. To test whether our findings were robust to the choice of 8 min catchments, we used sensitivity analyses based on six and 10 min catchments. In both cases, the findings were the same and there was no difference in the socioeconomic status of CFR and matched control catchments.

In addition, there were no data available on the numbers of active or on-duty volunteers for the CFRs, nor their exact residential location, which is likely their actual point of dispatch. This information may have helped to define the shape and extent of the CFR catchments more accurately. However, it would likely also have added substantially to the complexity of the analysis, without necessarily improving the accuracy of the outputs. In addition, it is important to note that the travel time data were derived from the OpenStreetMap routing machine, and were assumed to be representative for private car travel. If there is a bias in terms of the time of day at which OHCAs occur, then it is possible that driving conditions could be different to those described in the OSRM database. However, given that most of the CFRs are outside cities, there is unlikely to be a substantial impact from traffic congestion or other driving conditions that may impact the estimates of drive times.

Our analysis incorporates two measures of socioeconomic conditions, namely deprivation and social fragmentation. Social fragmentation was originally created as a measure of the non-economic aspects of deprivation (Congdon, 1996). It is a reverse measure of social capital and is intended to capture populations in flux and where there may be a lack of social cohesion. Hence, residents may invest less in the community as a result. Although deprivation and social fragmentation do not always coexist, they have previously been observed to be correlated, especially in studies involving big towns and cities (Area Based Analysis Unit (Office for National Statistics 1) (2009); Congdon, 2004). Therefore, the utility of the measure of social fragmentation in our study may be undermined by a correlation with deprivation. The validity and reliability of both the deprivation and social fragmentation measures is open to debate. Establishing the validity of a deprivation index is challenging (Beduk, 2018). Both indices are limited to a small number of variables with a direct link to the concept being quantified. The deprivation index has shown a consistency over time in terms of the classification of small areas, and has been used widely for health services research in Ireland (Teljeur et al., 2019).

A further limitation to our analysis is that it does not incorporate data on OHCA events. At a small-area level, OHCAs are rare events and subject to substantial variability from year to year. As a result, calculating an area-level risk of OHCA may not fully explain the distribution of CFRs but it might create the momentum needed to spur a community into action. While our focus was on the socioeconomic status of the catchment areas, it may be possible to consider the catchments in tandem with the socioeconomic status of OHCA cases and of the first responders themselves. The value of such an analysis would be to understand whether the responders represent the communities in which OHCAs are most likely to occur or the communities which are most likely to benefit from the provision of a CFR.

A final caveat is that we analysed the difference between the catchment socioeconomic status of CFRs and matched controls with increasing catchment size (in increments of 0.1 min). At each increment a Kolmogorov-Smirnov test was carried out to compare distributions. Arguably the repeated application of the test creates an issue with multiple hypothesis testing. We made no adjustment for this, although the findings are consistent with the tests run for catchment centroids and full catchment extent, and the purpose was to identify the point at which the difference in the distribution of socioeconomic scores is no longer important.

Overall, despite these limitations, this paper strongly suggests that a self-selection model for CFR recruitment does not disadvantage more deprived communities, though there is likely be a need for increased coverage of CFR schemes more generally. Community intervention is essential if we are to improve the rate of survival from OHCA. While OHCA incidence tends to be higher in more deprived areas and volunteerism is often higher in affluent areas, our findings show that those most at risk are not disadvantaged in terms of access to CFR schemes in Ireland and is a model that should be supported both in Ireland and other jurisdictions.

## Ethics statement

This study received ethical approval from the National University of Ireland Research Ethics Committee (Ref 18-Sep13).

## Funding

This work was supported by the 10.13039/501100001590Health Research Board, Ireland [grant number: APA-2016-1859]. This grant was paid to the 10.13039/100009770National University of Ireland Galway, and Dr Masterson is the Primary Investigator for this grant award.

## Author contributions

All authors contributed equally to conceptualisation of the research idea. Siobhán Masterson acquired funding, curated data and administered the project. Conor Teljeur designed the methodology and performed statistical analysis. John Cullinan performed validation of formal analysis. All authors contributed equally to writing the original draft, and subsequent review and editing.

## Declaration of competing interest

The authors can confirm that they have no conflicts of interest to declare.
