# Supplementary material for: Are there socioeconomic disparities in geographic accessibility to community first responders to out-of-hospital cardiac arrest in Ireland?
Source: SSM Popul Health. 2022 Jun 22;19:101151. doi: 10.1016/j.ssmph.2022.101151 (PMC9249950; doi:10.1016/j.ssmph.2022.101151)
Supplement: Multimedia component 1 [file mmc1.docx]

**Supplementary appendix**

Are there Socioeconomic Disparities in Geographic Accessibility to Community First Responders to Out-of-Hospital Cardiac Arrest in Ireland?

Siobhan Masterson, Conor Teljeur, John Cullinan

SSM – Population Health

This supplementary appendix outlines the propensity score matching models used, the explanatory variables included and the outputs of the balance checking criteria applied.

**Model specification**

In the main analysis, eight approaches to propensity score matching were assessed:

| Model | Method | Distance | Caliper |
| --- | --- | --- | --- |
| 1 | Nearest neighbour | logit | None |
| 2 | Nearest neighbour | logit | 0.10 |
| 3 | Nearest neighbour | logit | 0.25 |
| 4 | Nearest neighbour | Mahalanobis | None |
| 5 | Nearest neighbour | Mahalanobis | 0.10 |
| 6 | Nearest neighbour | Mahalanobis | 0.25 |
| 7 | Optimal search | logit | None |
| 8 | Optimal search | Mahalanobis | None |

**Explanatory variables**

Nine explanatory variables were included in the matching process:

| Abbreviated name | Description |
| --- | --- |
| minamb | Distance to nearest ambulance station |
| minhosp | Distance to nearest hospital with a 24/7 emergency department |
| mincity | Distance to nearest city |
| mintown | Distance to nearest town |
| minvilln | Distance to nearest village (close to an urban area) |
| minvillr | Distance to nearest village (remote from an urban area) |
| area | Geographic area of the catchment |
| pop | Population living in the catchment |
| pdens | Population density in the catchment |

**Results of balance checking criteria.**

Criterion 1: two-sample t-statistics between the mean of the treatment group and the mean of the control group for each explanatory variable.

A value of less than 0.05 indicates a statistically significant difference between the treatment and control group means. The optimal search approaches (models 7 and 8) are the only models that have statistically significant differences after matching.

| Variable | Model |  |  |  |  |  |  |  |
| --- | --- | --- | --- | --- | --- | --- | --- | --- |
|  | 1 | 2 | 3 | 4 | 5 | 6 | 7 | 8 |
| minamb | 0.950 | 0.966 | 0.803 | 0.976 | 0.974 | 0.945 | 0.904 | 0.976 |
| minhosp | 0.936 | 0.950 | 0.698 | 0.929 | 0.920 | 0.955 | 0.004 | 0.077 |
| mincity | 0.951 | 0.581 | 0.188 | 0.939 | 0.941 | 0.942 | 0.000 | 0.000 |
| mintown | 0.660 | 0.836 | 0.921 | 0.996 | 0.994 | 0.976 | 0.250 | 0.030 |
| minvilln | 0.845 | 0.584 | 0.764 | 0.879 | 0.887 | 0.891 | 0.000 | 0.000 |
| minvillr | 0.871 | 0.782 | 0.998 | 0.941 | 0.946 | 0.926 | 0.273 | 0.931 |
| area | 0.942 | 0.763 | 0.805 | 0.570 | 0.595 | 0.577 | 0.155 | 0.259 |
| pop | 0.296 | 0.838 | 0.780 | 0.270 | 0.270 | 0.262 | 0.327 | 0.149 |
| pdens | 0.574 | 0.832 | 0.718 | 0.333 | 0.333 | 0.327 | 0.648 | 0.083 |

Criterion 2: mean difference as a percentage of the average standard deviation.

Low values indicate that the difference in treatment and control means is small relative to the average standard deviation of the treatment and control groups. The lowest differences are observed for the three models based on Mahalanobis distance and nearest neighbour matching (models 4, 5, and 6).

| Variable | Model |  |  |  |  |  |  |  |
| --- | --- | --- | --- | --- | --- | --- | --- | --- |
|  | 1 | 2 | 3 | 4 | 5 | 6 | 7 | 8 |
| minamb | 82.1 | 88.3 | 84.9 | 11.2 | 11.2 | 11.3 | 79.9 | 83.7 |
| minhosp | 95.5 | 97.2 | 88.1 | 11.6 | 11.4 | 11.6 | 101.4 | 105.1 |
| mincity | 93.1 | 101.3 | 97.5 | 9.2 | 9.1 | 9.1 | 155.9 | 149.5 |
| mintown | 83.9 | 92.5 | 81.6 | 9.8 | 9.9 | 9.8 | 82.5 | 84.5 |
| minvilln | 76.8 | 80.6 | 82.6 | 10.2 | 10.3 | 10.2 | 109.7 | 108.7 |
| minvillr | 89.3 | 85.2 | 82.6 | 14.5 | 14.0 | 13.9 | 85.0 | 92.9 |
| area | 77.1 | 79.9 | 78.0 | 26.7 | 26.6 | 27.0 | 72.8 | 120.8 |
| pop | 50.5 | 51.5 | 52.3 | 23.2 | 23.2 | 23.3 | 44.2 | 66.5 |
| pdens | 37.4 | 41.0 | 39.2 | 19.2 | 19.2 | 19.2 | 35.2 | 63.0 |

Criterion 3: percentage reduction of bias in the means of the explanatory variables after and before matching

A large negative value suggests a large reduction in the difference between treatment and control group means relative to the difference in means before matching. A positive value indicates an increase in bias. The largest average reductions were achieved by the three models based on Mahalanobis distance and nearest neighbour matching (models 4, 5, and 6) and the model based on Mahalanobis distance and optimal search (model 8).

| Variable | Model |  |  |  |  |  |  |  |
| --- | --- | --- | --- | --- | --- | --- | --- | --- |
|  | 1 | 2 | 3 | 4 | 5 | 6 | 7 | 8 |
| minamb | -88.7 | -88.7 | -88.7 | -88.4 | -90.7 | -90.7 | -78.2 | -87.0 |
| minhosp | -93.3 | -93.3 | -93.3 | -95.2 | -92.1 | -92.1 | 135.4 | -92.8 |
| mincity | -89.2 | -89.2 | -89.2 | -82.9 | -83.4 | -83.4 | 2357.7 | -87.7 |
| mintown | -0.7 | -0.7 | -0.7 | -94.2 | -96.2 | -96.2 | 159.3 | -94.1 |
| minvilln | -69.6 | -69.6 | -69.6 | -67.5 | -71.5 | -71.5 | 604.4 | -73.1 |
| minvillr | -87.7 | -87.7 | -87.7 | -92.6 | -94.0 | -94.0 | -19.0 | -93.5 |
| area | -97.3 | -97.3 | -97.3 | -81.0 | -81.0 | -81.0 | -49.3 | -82.2 |
| pop | -72.7 | -72.7 | -72.7 | -72.5 | -71.2 | -71.2 | -71.2 | -72.7 |
| pdens | -86.4 | -86.4 | -86.4 | -78.6 | -78.0 | -78.0 | -87.7 | -78.7 |

Criterion 4: Kolmogorov-Smirnov test to compare the treatment and control group density estimates for each explanatory variable

A value of less than 0.05 indicates a statistically significant difference between the treatment and control group density estimates for a given explanatory variable. Models based on Mahalanobis distance and nearest neighbour matching (models 4, 5, and 6) had the fewest statistically significant differences after matching.

| Variable | Model |  |  |  |  |  |  |  |
| --- | --- | --- | --- | --- | --- | --- | --- | --- |
|  | 1 | 2 | 3 | 4 | 5 | 6 | 7 | 8 |
| minamb | 0.075 | 0.226 | 0.274 | 0.978 | 0.978 | 0.978 | 0.329 | 0.459 |
| minhosp | 0.274 | 0.075 | 0.459 | 0.999 | 0.993 | 0.999 | 0.000 | 0.000 |
| mincity | 0.459 | 0.003 | 0.533 | 0.978 | 0.978 | 0.978 | 0.000 | 0.000 |
| mintown | 0.000 | 0.000 | 0.000 | 0.390 | 0.329 | 0.329 | 0.006 | 0.011 |
| minvilln | 0.008 | 0.120 | 0.075 | 0.902 | 0.902 | 0.902 | 0.000 | 0.000 |
| minvillr | 0.184 | 0.184 | 0.611 | 0.769 | 0.769 | 0.769 | 0.006 | 0.226 |
| area | 0.390 | 0.769 | 0.978 | 0.948 | 0.948 | 0.902 | 0.149 | 0.390 |
| pop | 0.001 | 0.011 | 0.008 | 0.004 | 0.004 | 0.003 | 0.000 | 0.001 |
| pdens | 0.000 | 0.004 | 0.004 | 0.001 | 0.001 | 0.000 | 0.000 | 0.000 |
